# Cocaine-induced Thrombosis: Review of Predisposing Factors, Potential Mechanisms, and Clinical Consequences with a Striking Case Report

**DOI:** 10.7759/cureus.4700

**Published:** 2019-05-21

**Authors:** Toishi Sharma, Manish Kumar, Alain Rizkallah, Erika Cappelluti, Premkumar Padmanabhan

**Affiliations:** 1 Internal Medicine, Hartford Hospital, Hartford, USA; 2 Internal Medicine, University of Connecticut Health Center, Farmington, USA; 3 Cardiology, Hartford Hospital, Hartford, USA

**Keywords:** cocaine, cocaine related stroke, cocaine thrombosis, pulmonary embolism, bilateral pulmonary embolism, deep vein thrombosis, drug abuse

## Abstract

Cocaine is associated with a wide array of complications through a number of different mechanisms. Although the majority of cocaine-related morbidity has been attributed to complications in arterial vasculature, the deleterious impact of venous complications appears to be largely unrepresented in current literature as well as clinical practice despite emerging evidence of the high prevalence and annual incidence of deep vein thrombosis (DVT) in illicit drug users.

Our case report illustrates an uncharacteristic presentation of cocaine-related widespread thrombotic cascade involving both arterial and venous circulations causing significant morbidity. The complex pathophysiology of widespread prothrombotic state caused by cocaine includes endothelial damage promoting the increase of fibrinogen and Von Willebrand factor to platelet aggregation and clot formation. It is important to identify the impact cocaine-induced venous thrombosis can mount, especially in the form of potentially fatal complications like pulmonary embolism. Although recent studies have focused on increased incidence and prevalence of venous thrombosis in the setting of cocaine abuse, ours is the first case of a documented pulmonary embolism caused by cocaine-related venous thrombosis. Further studies are needed to identify patients at higher risk for this complication like rare thrombotic disorders.

## Introduction

Majority of drug misuse emergency department (ED) visits nationwide have been contributed by cocaine use. Additionally, short term visits involving illicit drugs were documented to have experienced an increase by a tremendous 29% [1-2].

The consumption of cocaine is associated with a wide array of complications through a number of different mechanisms. These adverse effects include a wide range of cardiac [3], arterial dissections [3-5] as well as cerebrovascular complications [6-7]. Although a vast majority of cocaine-related morbidity has been attributed to complications involving arterial vasculature, the deleterious impact of venous complications appears to be largely unrepresented in current literature as well as clinical practice despite emerging evidence of the high prevalence and annual incidence of deep vein thrombosis (DVT) in illicit drug users compared to the general population [8].

This case illustrates an uncharacteristic presentation of cocaine-related widespread thrombotic cascade involving both arterial and venous circulations and causing significant morbidity.

## Case presentation

A 27-year-old previously healthy male presented to the ED with a new onset of difficulty speaking and facial droop. The patient was last seen normal eight hours prior to the presentation when he went to sleep. On waking up, his brother noticed a slurred speech along with a right facial droop and called the emergency medical services.

On arrival, he was afebrile with the following vitals: temperature 98.5 °F, blood pressure 142/97, heart rate 94, and respiratory rate 16. The examination was remarkable for expressive aphasia and right facial droop with no other focal neurological finding. He was alert and oriented to person, place, time and in no acute distress apart from being irritable due to word finding difficulty. History and review of symptoms were limited due to the patient’s aphasia, and writing board was used to illicit limited pertinent history. He denied headache, nausea, vision changes, weakness, chest pain or palpitations. Although initially hesitant, he eventually reported using intravenous heroin on a daily basis and cocaine for the first time on the previous day. Family history, as reported by his mother, was remarkable for death of patient’s father from a myocardial infarction (MI) three months ago, history of MI in his uncle at age of 46, and two spontaneous abortions in paternal aunt who had a known clotting disorder, the specifics of which could not be established.

**Figure 1:**
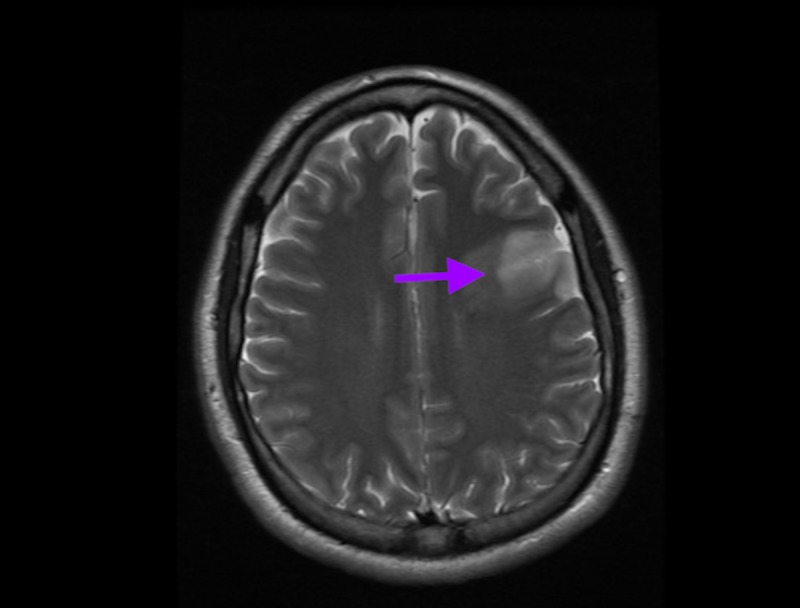
Brain imaging showing acute frontal infarct

**Figure 2:**
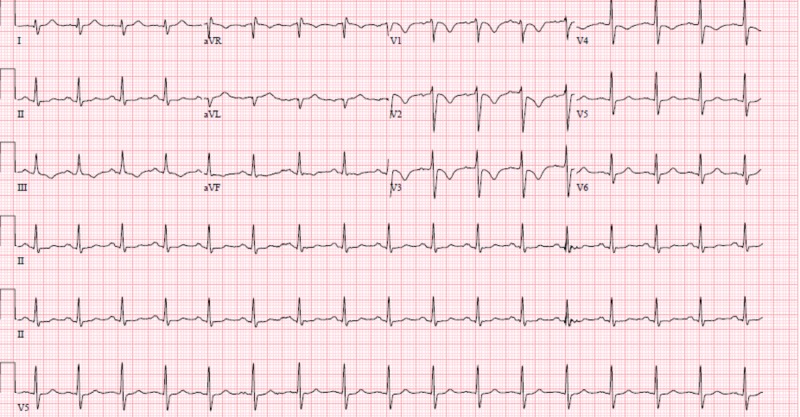
Electrocardiogram (EKG) with T-wave inversions in the anterior and inferior leads suggesting ischemia

Stat computed tomography (CT) scan of the head showed thrombozed left middle cerebral artery (MCA) along with infarct in the left frontal lobe as well as basal ganglia (Figure 1). Hematological labs were significant only for a troponin of 14 md/dl and CK-MB 34. Other labs including complete blood counts (CBC) and a comprehensive metabolic panel were unremarkable. Urine toxicology returned positive for cocaine and opiates. Electrocardiogram (EKG) showed T-wave inversion (TWI) with ST depression in V1-V4, TWI in II and arteriovenous fistula (AVF) with right axis deviation (Figure 2).

**Figure 3:**
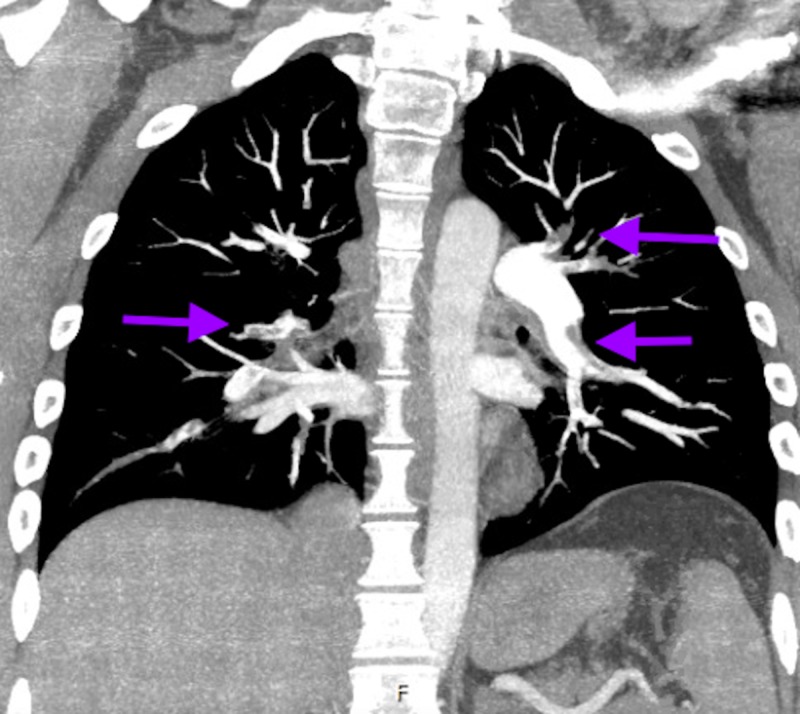
Computed tomography angiography (CTA) of the chest showing bilateral segmental embolism

**Figure 4:**
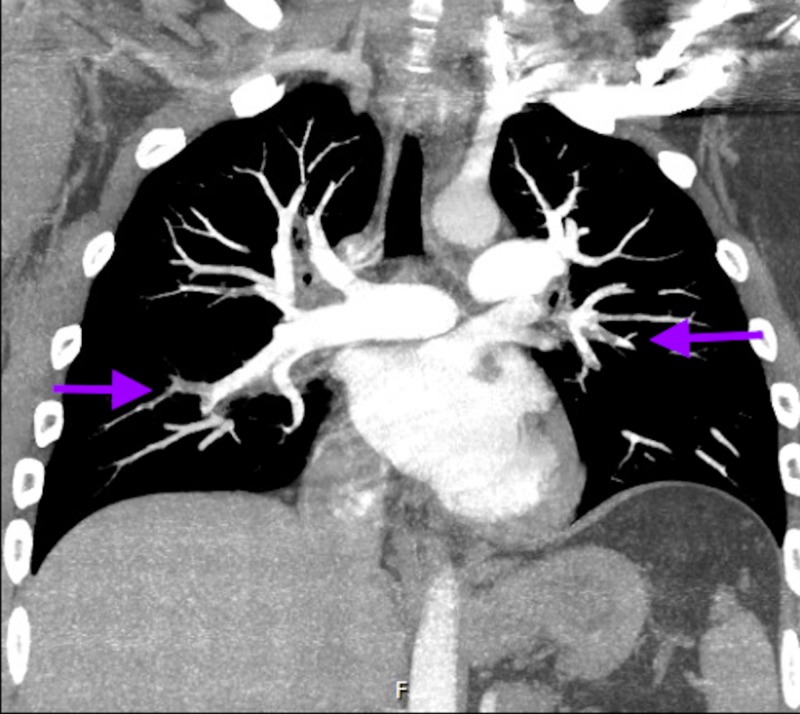
Computed tomography angiography (CTA) of the chest showing subsegmental embolism

Computed tomography angiography (CTA) of head and neck showed complete abrupt occlusion of the proximal left internal carotid artery (ICA) with distal reconstitution at the segment M1 of MCA and an intraluminal thrombus in the M1 segment. An ICA dissection leading to occlusion was possible as per the imaging. A limited bedside echocardiogram was remarkable for severely reduced right ventricle systolic function with akinesia of basal and mid free wall raising concern for right ventricular infarct versus strain from a pulmonary embolism (PE). CTA of chest done due to significant troponinemia and EKG/echo findings revealed bilateral segmental and sub-segmental PE without any acute pulmonary disease (Figures 3-4).

In the setting of acute stroke, the patient was deemed not a candidate for tissue plasminogen activator (tPA) or endovascular intervention due to low National Institutes of Health Stroke Scale (NIHSS) scale of 4. At presentation, he was also not considered a candidate for percutaneous coronary intervention or anticoagulation, due to high risk of hemorrhagic conversion of his territorial stroke should he require dual antiplatelet therapy and/or anticoagulation if found to have coronary artery thrombosis.

Further management included placing an inferior vena cava filter in setting on PE with contraindication to anticoagulation. Magnetic resonance imaging (MRI) of the brain showed infarction in the left frontal lobe and basal ganglia consistent with patient’s expressive aphasia as well as small areas of deep cortical infarction in the caudate and sub-insular area. A trace area of hemorrhagic transformation was identified in the caudate region. A later Doppler of lower extremities revealed DVT in the left peroneal and posterior tibial veins.

Due to limited area of hemorrhagic transformation identified in the caudate region, after the multidisciplinary team’s discussion with the family, a decision was made to start the patient on low dose anticoagulation without a bolus with plan to repeat a CT head over the next 24 hours to monitor for worsening hemorrhagic conversion while being continued on Q1 hour neuro-checks.

A following comprehensive echocardiogram with a bubble study next day failed to reveal an intracardiac shunt precluding the possibility of paradoxical thrombosis. While still awaiting the results of hypercoagulable workup and repeat CT head without worsening hemorrhagic conversion, the patient was continued on anticoagulation with intravenous heparin for 10 days before being switched to oral anticoagulant Xarelto. He experienced improvement in word finding ability with every passing day and was beginning to frame two to three-word phrases by the end of 10 days when his care was transitioned to a rehabilitation facility for continued physical, occupational, and speech therapy. Subsequently, hypercoagulability work-up including prothrombin gene mutation, factor V Leiden deficiency, anti-phospholipid antibody (APLA), and anti-thrombin deficiency eventually turned out to be negative.

## Discussion

Right from the initial presentation, it was challenging to find a unifying diagnosis for this peculiar constellation of findings including widespread venous and arterial thrombosis causing DVT and PE, carotid dissection, and ischemic stroke. However, the initial leading hypothesis was that an intracardiac shunt, if present, would explain the paradoxical thrombus shower into arterial circulation including coronary arteries as well as ICA. Given the patient's young age with a significant family history of MIs, pregnancy loss and known clotting disorder, the genetic hypercoagulable state was also high on differential; appropriate tests for factor 5 Leiden mutation, APLA, prothrombin gene mutation, and antithrombin were conducted. Although low on differential, there was a cascade of thrombosis caused by intravenous drug abuse leading to arterial as well as venous thrombosis.

However, with subsequent comprehensive echocardiogram unrevealing for a patent foramen ovale and negative for common hypercoagulable states, left a few possibilities which could include rare thrombotic states (sticky platelet syndrome, protein C, homocystinemia, and protein S deficiency), acute hypercoagulability caused by illicit drug abuse or possibly, a combination of both.

Cocaine-related complications, especially in the arterial circulation have been well documented These include cardiac complications such as sudden death, acute myocarditis, dilated cardiomyopathy, life-threatening arrhythmias, myocardial ischemia and infarction, dissections of coronary, aortic, carotid, and arteries. The major cerebrovascular effects of cocaine consist of ischemic and hemorrhagic strokes [6-7]. The mechanisms of these complications have been well studied (Table 1). However, it is interesting to note that a pathophysiological process is common to all the above-mentioned categories of vascular complications. This is the widespread prothrombotic state caused by cocaine-induced accelerated thrombosis, the complex pathophysiology of which can be summarized as follows: cocaine ingestion causing acute stress leads to endothelial damage promoting increase of fibrinogen (glycoprotein, which helps in formation of blood clots) and Von Willebrand factor (glycoprotein signaling endothelium changes), leading to platelet aggregation and ultimately the formation of blood clots [9].

**Table 1:** Mechanisms implicated in cocaine-mediated vascular complications

Mechanisms implicated in cocaine-mediated vascular complications		
Category	Adverse effects	Proposed mechanisms till date
Cardiac	Sudden death, acute myocarditis, dilated cardiomyopathy, life-threatening arrhythmias, myocardial ischemia & infarction	combination of cocaine-induced vasospasm through adrenergic stimulation, intracoronary thrombosis triggered by alterations in the plasma constituents, and platelet aggregation
Dissections	Coronary, aortic, carotid and renal artery dissection	Mainly accounted by cocaine-mediated hypertension, recent preclinical studies have postulated that cocaine may cause apoptosis of vascular endothelial and/or smooth muscle cells, thus weakening the vascular wall and resulting in a dissection-prone state
Neural	Ischemic and hemorrhagic strokes, sub-arachnoid hemorrhage and hypertensive encephalopathy	Acute hypertension, endothelial dysfunction and vascular injury, a prothrombotic state, impaired cerebral blood flow, and cerebral artery vasoconstriction induced by cocaine’s sodium-blocking effect

Additionally, inflammation and atherosclerosis are substantial potentially lethal vascular effects of cocaine use that have acute and chronic systemic impact [10-13] Cocaine also creates an elevated immune system inflammatory state with decreased basal anti-inflammatory markers (e.g., interleukin-10) [11] and increased pro-inflammatory cytokines (e.g., tumor necrosis factor alpha, Interleukin 1β) all contributing to vascular disease (e.g., endocarditis) [1-15].

In contrast to arterial thrombosis, the prevalence, incidence, and morbidity associated with cocaine-induced venous thrombosis seem to be overlooked in the current literature. This is likely due to the acuity, higher frequency, and mortality associated with arterial thrombosis. However, as exemplified in our case report, it is important to identify the impact cocaine-induced venous thrombosis can mount, especially in the form of potentially fatal complications like pulmonary embolism. Although ours is the first case of a documented pulmonary embolism caused by cocaine-related venous thrombosis, many recent studies have focused on increased incidence and prevalence of venous thrombosis in the setting of cocaine abuse [16].

Although the mechanisms of clotting used to explain arterial thrombosis can be implicated in venous thrombosis as well, the lower prevalence of the latter prompts further curiosity in elucidating additional factors that may be involved in pathogenesis cocaine-related venous thrombosis; the identification of these factors is crucial for their anticipation and management. Postulated mechanisms include direct toxicity of cocaine to the veins possibly exacerbated by the citric acid used to dissolve the drugs and the local anesthetic property of the cocaine [17], adulteration of cocaine with vasoactive substances such as quinine, procaine phencyclidine, antihistamine, methamphetamine [18], reduced blood flow from inactive muscle pumps during periods of intoxication, endothelial damage from injections, and elevated coagulation factors from infection introduced via injections [19-20].

## Conclusions

Cocaine can cause not only arterial but also significant venous thrombosis via multiple possible mechanisms and this can contribute to significantly increased morbidity associated with cocaine abuse. As the stride into identifying the most potent risk factors for cocaine-induced venous thrombosis continues, a clinical pearl which can be elucidated from this case is that cocaine abuse in a patient with a family history of clotting disorders can lead to devastating outcomes. Anyone with a personal or family history of thrombotic disorders should be particularly warned of potentially fatal complications of cocaine abuse; and in those presenting with it, it may be befitting to chase rare genetic disorders that still need to be identified to better serve this population.
